# Hæmorrhoids

**Published:** 1882-10

**Authors:** A. McNeil

**Affiliations:** Jeffersonville, Ind.


### CLINICAL BUREAU.

### HÆMORRHOIDS.

A. McNeil, M. D., Jeffersonville, Ind.

November 30th, 1881, Mrs. H., æt. 51, has passed the change of life. The expression on her face revealed the most intense suffering. She is sallow, medium flesh. Had piles several years ago which have not troubled her till three weeks ago; are getting worse all the time. She says she did not suffer as excruciatingly in childbirth as she has been doing since this attack. It feels, she says, as if a red-hot poker was being thrust up the rectum. She is temporarily relieved by sitting in cold water, and by sitting on her foot so as to press on the anus; bowels loose; profuse discharge of blood.

Guided by relief by sitting on her foot, I gave her Kali carb.$^{30}$ every three hours. Bœnninghausen recommends the Kali carb. in piles relieved by riding on horseback, which means that violent pressure relieves.

December 2d. Pains have subsided so that she says that she is comfortable. Sac. lac.

December 9th. Was very comfortable till yesterday. "Caught cold," causing a slight return of piles and some drawing in the nape of neck. Kali carb.$^{m}$ one dose.

December 19th. No piles; same stiffness in neck. Has an old difficulty of the heart; cannot lie on right side or with her head low. Kali carb.$^{20m}$, in ten powders every four hours. She was then well enough to go to Little Rock, Ark. Before going she told me that she had been induced to try me on the recommendation of a neighbor, who told her that I had cured him and his brother of piles in a very short time, but I had not kept notes of their cases.

Can ligation, cutting, or injection of Carbolic acid cure piles *tuto cito et jucunde?* I boldly say, No! And when they do succeed with those heroic measures in relieving the hæmorrhoids it is usually by transferring the disease to a vital organ. "The piles were cured but the patient died."

The most advanced minds of the old school are beginning to find out that Hahnemann was right when he said that piles, eruptions, ulcerations and displacements of the womb, etc., are but local manifestations of constitutional diseases. But so-called liberal homœopaths will pick up the cast-off clothes of our allopathic colleagues and strut proudly in the "physiological livery."

Lichen ruber. August 18th, 1881, Lizzie Smith, æt. 15. Has had this eruption four years, during which it varied in intensity and sometimes disappeared. It occupies both arms, extending on to the backs of the hands. It is bright red and hot to the touch; does not itch, but feels prickly. Has had urticaria once, is subject to headache, all of which are relieved by cold water applications. Sometimes the bowels are covered and have crusts on them. When a child she had a disease in her hip which was diagnosed as hip disease by specialists. My diagnosis was only made after a careful study of the case and a comparison of the eruption with Baerensprung und Hebra's Atlas der Hautkranhieten Tafel.

I gave her Apis$^{30}$, ten powders every twenty-four hours.

August 24th. Redness all gone, only occasionally an uncomfortable sensation, but is worse to-day. Apis$^{30}$, ten powders every four hours.

September 10th. Has been well until to-day, when the prickling returned in the palms of both hands. Apis$^{20m}$, one powder.

September 22d. Has been well until a day or two ago. Has now a few scattering pimples, the remains of urticaria wheals. Apis$^{20m}$, in one dose. Remained well till in January, when I last saw her.

Was this case cured or did it get well? Let us see. Duhring says, *Diseases of the Skin*, 2d edition, page 243: "The sooner in the course of the disease treatment is instituted, the more speedy will be the cure; cases of long standing are found to be exceedingly obstinate, continuing for long periods but slightly influenced by remedies which in an earlier stage would have afforded relief."

Four years' continuance would, I venture to say, place this case among cases of long standing, and yet it was not "found to be exceedingly obstinate" to the indicated homœopathic remedy in the minimum dose. Duhring does not hint that such cases as mine get well of themselves. But let us see what a greater than Duhring says, Hebra *Hautkrankheiten*, von Hebra und Kaposi, Band I, page 397:

"We must truthfully say in sincere sorrow that no medicine, either locally applied or internally administered, with the exception of Arsenic has made any essential change in the course of this disease."

"On the other hand, Arsenic has in no case left us in the lurch in which it was given in sufficient quantity. As long as we did not have the courage to give sufficiently large doses or the perseverance to administer small doses for a long enough time, we had no favorable results to record. See the 1st edition of our work, page 3. However, since we have been taught by experience how to use it [abuse? Trans.] we have had only pleasing effects to report. To obtain such beneficial results it is necessary to give the Arsenic persistently for many—six to eighteen—months. In this way we have given some of our patients, among them children of twelve to thirteen years, in a period of a few months or a year, as many as 3,500 Asiatic pills, *i. e.*, 250 grains of white Arsenic, curing the patients and restoring the nutrition of their bowels to the normal."

Is not this case worth a thousand Milwaukee tests? And who would not prefer curing with an infinitesimal in one month to taking 250 grains of Arsenic in the course of eighteen?

---

A CHILD, æt. six years, while throwing stones at a toad, suddenly felt the animal spurt some moisture into his eye. There suddenly set in a slight pain and spasmodic twitching of the injected eye; two hours after, coma, jumping sight, desire to bite, dread of food and drink, constipation, abundant urine, and great agitation manifested themselves, followed on the sixth day by sickness, apathy, and a kind of stupor, but with a regular pulse. Some days later, having become comparatively quiet, he left his bed; his eyes were injected, the skin dry, the pulse free from fever.

He howled and behaved like a madman, sank into imbecility and speechlessness, from which he never rallied.—*Phila. Med. and Surg. Reporter, Dec.* 14*th*, 1878.

